# A Facile Synthesis of 2,4-Disubstituted Thiazoles Using MnO_2_

**DOI:** 10.3390/molecules14124858

**Published:** 2009-11-26

**Authors:** Yan-Bo Yu, Hong-Liang Chen, Li-Yi Wang, Xin-Zheng Chen, Bin Fu

**Affiliations:** Department of Applied Chemistry, China Agricultural University, 100193, Beijing, China; E-Mails: yuyanbo0902@163.com (Y.-B.Y.); chenhongliang1016@yahoo.cn (H.-L.C.); Ritaxiaoyi@163.com (L.-Y.W.); xinzheng1943@126.com (X.-Z.C.)

**Keywords:** thiazole, thiazoline, manganese dioxide, oxidation

## Abstract

Structurally diverse thiazoles with electron-donating and electron-withdrawing groups were conveniently synthesized through manganese dioxide (MnO_2_) oxidation of the corresponding thiazolines. The effect of substitution at the 2- and 4-positions was investigated. The desired thiazoles with aryl or vinyl substitutions at the 2- or 4-position can be obtained in good to excellent yields.

## 1. Introduction

The thiazole ring is an interesting building block in a variety of natural products and bioactive compounds useful as pharmaceuticals or agrochemical agents [1,2,3,4,5], and to date many methods have been developed for the construction of thiazole ring systems. One classical and widely used method is the condensation of α-haloketones with thioamide derivatives, which is known as the Hantzsch reaction [6,7,8]. Another efficient method is the introduction of substitutions onto a thiazole core structure through Stille coupling [9], which involves the use of organostannane intermediates. In recent years, a new and frequently encountered method for thiazole synthesis is the conversion of thiazoline derivatives through the use of dehydrogenating reagents such as sulfur [10], KMnO_4_ [11], Cu(I)/Cu(II)/peroxide oxidation [12], MnO_2_ [13,14,15,16], NaH/DBU [17], and so on. Among these dehydrogenating reagents, activated MnO_2_ is a very simple and convenient reagent for the synthesis of thiazoles from thiazolines. However, all cases of MnO_2 _oxidation of thiazolines reported in the literature are restricted to thiazoles bearing electron-withdrawing substituents such as carboxylates, and to the best of our knowledge, no report involving the use of MnO_2_ for the synthesis of thiazoles without carboxylate substitution has appeared. To investigate the generality and scope of this method as a continuation of our research interest in thiazoline chemistry [18,19,20], we would like to report the synthesis of 2,4-disubstituted thiazoles with electron-donating and electron-withdrawing groups from the corresponding thiazolines *via* activated MnO_2_ oxidation.

## 2. Results and Discussion

**Scheme 1:**
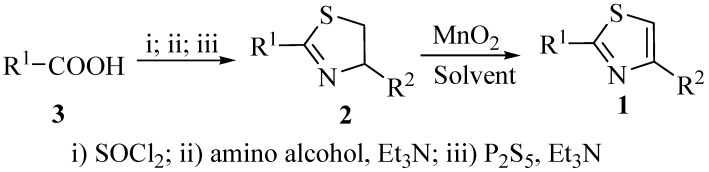
The synthesis of 2,4-disubstituted thiazoles.

The starting thiazolines **2** were easily prepared in one-pot reactions from the corresponding carboxylic acids **3** or their derivatives [19,20], and commercially available amino alcohols which provide R^2^ in the product (Scheme 1). 

**Scheme 2:**
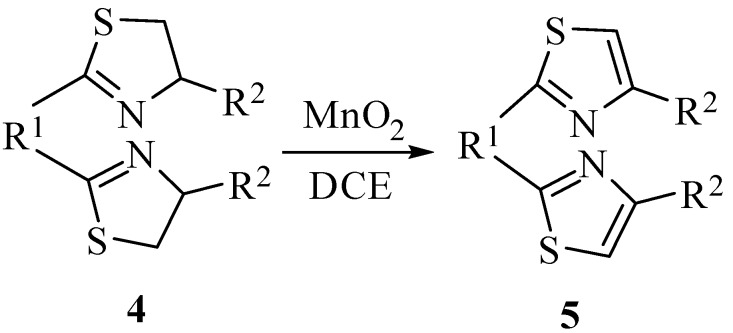
The synthesis of 2,4-disubstituted bis-thiazoles.

With all kinds of thiazoline derivatives in hand, we first set out to optimize the reaction conditions. The suspension of thiazoline **2a** and excess activated MnO_2_ (10 equiv.) in dichloromethane (DCM) was stirred for 24 h at room temperature [15,16]. No desired product was yielded even the reaction temperature was elevated to the boiling point for 48 hours. Through extensive screening of solvents, we observed that the reaction proceeded well under reflux in solvents with different polarity but similar boiling points. The results indicated the strong correlation between the yield and the reaction temperature. In DCE, CH_3_CN, or benzene, full conversion and up to 95% isolated yields can be achieved within 12 hours. In the case of toluene, the starting material disappeared within 6 h and the thiazole product was afforded in 80% yield. Lowering the ratio of oxidant to thiazoline led to the significant decrease of the reaction rate. With the optimized condition in hand (DCE as solvent, 1:10 molar ratio of thiazoline to MnO_2_), thiazoles with different substitutions at the 2- and 4-positions were synthesized (Table 1). In most cases, the reaction proceeded well under reflux (entries 6–16). When one of 2- and 4-position of thiazoline is an aryl or vinyl group, the thiazole products are produced in good to excellent yields (entries 6, 7, 9, 10, 11, 14 and 16). When both the 2- and 4-position of thiazoline are aryl groups, the yields were improved to 95%–99 % (entries 8, 12 and 13), which can be ascribed to the stronger conjugation effect between aryl groups and thiazoles. In contrast, when both 2- and 4-positions of thiazoline are alkyl groups, none of the desired thiazole products was obtained (entry 17). The scope of this method was further exploited to the preparation of bis-thiazoles (Scheme 2). The desired products were also obtained in high yield from corresponding bis-thiazolines, as illustrated in Table 2 (entries 1–4).

**Table 1:** The conversion of thiazolines to thiazoles by MnO_2_ oxidation^a^.

Entry	Compd.	R^1^	R^2^	Solvent	Time(h)	Yield(%)
1	**1a**	Ph	Me	DCM	48	–
2	**1a**	Ph	Me	DCE	12	95
3	**1a**	Ph	Me	Benzene	12	90
4	**1a**	Ph	Me	CH_3_CN	12	90
5	**1a**	Ph	Me	Toluene	6	80
6	**1b**	Ph	*i*-Pr	DCE	12	90
7	**1c**	Ph	*i*-Bu	DCE	12	90
8	**1d**	Ph	Ph	DCE	12	99
9	**1e**	2-Py	Me	DCE	12	90
10	**1f**	2-Py	*i*-Pr	DCE	12	77
11	**1g**	2-Furyl	Bn	DCE	12	70
12	**1h**	2-Furyl	Ph	DCE	12	95
13	**1i**	2-thienyl	Ph	DCE	12	95
14	**1j**	PhCH=CH-	*i*-Pr	DCE	12	80
15	**1k**	PhCH=CH-	Ph	DCE	12	95
16	**1l**	Me	Ph	DCE	12	76
17	**1m**	Me	*i*-Pr	DCE	24	–

^a^ The reactions were run under reflux in different solvents.

**Table 2:** The conversion of bis-thiazolines to bis-thiazoles by MnO_2_ oxidation^a^.

Entry	Compd.	R^1^	R^2^	Reaction time	Yield
1	**5a**	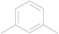	*i*-Pr	12	80
2	**5b**	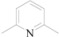	Me	12	85
3	**5c**	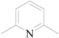	*i*-Pr	6	80
4	**5d**	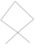	Ph	8	70

^a^ The reactions were run under reflux in DCE.

## 3. Conclusions

In conclusion, we have demonstrated that thiazoles bearing different electron-donating and electron-withdrawing groups can be conveniently synthesized from the corresponding thiazolines using activated MnO_2_ in dichloroethane. The critical effects of the reaction temperature and the substitutions on the thiazoline ring were investigated. The scope of this method was further extended to the preparation of 2,4-disubstituted thiazoles with diverse groups.

## 4. Experimental

NMR spectra were recorded on a Bruker Avance DPX300 spectrometer with tetramethylsilane as internal standard and CDCl_3_ as solvent. Infrared spectra were obtained on a Nicolet AVATAR 330 FT-IR spectrometer. Elemental analyses were carried out on an Elementar Vario EL instrument. Melting points were measured on an XT-4 melting point apparatus and were uncorrected. Solvents were purified and dried following standard procedures. 

### 4.1. Synthesis of Thiazolines

All thiazolines were prepared according to the literature [19,20].

### 4.2. Typical Procedure for Oxidation of Thiazolines to Thiazoles

To a solution of 4-methyl-2-phenylthiazoline (177 mg, 1 mmol) in 1,2-dichloroethane (10 mL) was added activated MnO_2_ (860 mg, 10 mmol). The mixture was then refluxed for 12 h under a nitrogen atmosphere. After filtration, the mixture was evaporated in *vacuo*. The residue was chromatographed on silica gel (ethyl Acetate-hexane, 10:1) to give 176 mg (95% yield) of 4-methyl-2-phenylthiazole (**1a**) [21] as a colorless oil; ^1^H-NMR: δ 7.94–7.91(m, 2H), 7.43–7.39 (m, 3H), 6.85 (t, *J* = 0.96 Hz, 1H), 2.50 (d, *J* = 0.96 Hz, 3H); ^13^C-NMR: *δ* 167.44, 153.71, 133.72, 129.65, 128.75, 126.34, 113.30, 17.14.

### 4.3. Spectral Data of Other Thiazole Compounds

***1b* [11]**: ^1^H-NMR: *δ* 7.96–7.91(m, 2H, ArH), 7.44–7.37 (m, 3H, ArH), 6.86 (s, 1H), 3.21–3.11 (m, 1H), 1.35 (d, *J*= 6.90 Hz, 6H); ^13^C-NMR: *δ* 167.29, 164.87, 134.08, 129.63, 128.79, 126.52, 110.88, 31.05, 22.40.

***1c***: colorless oil; IR (KBr, cm^−1^): 3063, 2955, 2928, 1516, 1461, 1244, 763; ^1^H-NMR: δ 7.95–7.91 (m, 2H, ArH), 7.49–7.35 (m, 3H, ArH), 6.85 (d, *J*= 0.63 Hz, 1H), 2.67 (dd, *J* = 9.0, 0.75 Hz, 2H), 2.16–2.06 (m, 1H), 0.97 (d, *J*= 6.60 Hz, 6H); ^13^C-NMR: *δ* 167.16, 157.77, 133.93, 129.57, 128.74, 128.43, 113.45, 40.78, 28.38, 22.35; Anal. Calcd. for C_13_H_15_NS (217.34): C 71.84, H 6.96, N 6.44. Found: C 71.96, H 6.85, N 6.23.

***1d* [22]**: white solid, mp: 90.5 °C–92.0 °C (lit. [22] 91.0–92.0°C);^ 1^H-NMR: *δ* 8.05–7.98 (m, 4H), 7.47–7.42 (m, 6H), 7.41–7.34 (m, 1H); ^13^C-NMR: *δ* 167.74, 156.21, 134.48, 133.72, 129.53, 128.83, 128.65, 128.08, 126.54, 126.34, 112.54.

***1e* [23]**: white solid, mp: 85.0–86.0 °C (lit. [23] 84.0–84.5 °C); ^1^H-NMR: *δ* 8.60–8.58 (m, 1H), 8.18–8.14 (m, 1H), 7.79–7.73 (m, 1H), 7.30–7.26 (m, 1H), 6.99 (d, *J* = 0.84 Hz, 1H), 2.52 (d, *J* = 0.84 Hz, 3H); ^13^C-NMR:*δ* 167.92, 153.81, 151.12, 149.06, 136.53, 123.87, 119.17, 115.84, 16.96.

***1f***: colorless oil; IR (KBr, cm^−1^): 3060, 2920, 1738, 1365, 1217; ^1^H-NMR: *δ* 8.60–8.58 (m, 1H), 8.21–8.18 (m, 1H), 7.79–7.73 (m, 1H), 7.29–7.25 (m, 1H), 6.98 (d, *J*= 0.84 Hz, 1H), 3.19–3.14 (m, 1H), 1.36 (d, *J* = 6.90 Hz, 6H); ^13^C-NMR: *δ* 167.96, 165.07, 151.61, 149.26, 136.71, 124.01, 119.57, 113.45, 30.96, 22.31; Anal. Calcd. for C_11_H_12_N_2_S (204.30): C 64.67, H 5.92, N 13.71. Found: C 64.88, H 5.91, N 13.45.

***1g***: colorless oil; IR (KBr, cm^−1^): 3120, 1569, 1495, 1473, 1299, 1133, 810, 769; ^1^H-NMR: *δ* 7.49 (t, *J* = 1.20 Hz, 1H), 7.35–7.22 (m, 5H, ArH), 6.97 (dd, *J* = 2.1, 0.6 Hz, 1H), 6.69 (s, 1H), 6.51 (dd, *J* = 4.80, 3.33 Hz, 1H), 4.17 (s, 2H); ^13^C-NMR: *δ* 157.81, 151.51, 149.04, 143.41, 138.89, 129.08, 128.54, 126.48, 113.59, 112.08, 108.79, 37.91; Anal. Calcd. for C_14_H_11_NOS (241.32): C 69.68, H 4.59, N 5.80. Found: C 69.75, H 4.85, N 5.93.

***1h***: white solid, mp: 72.3–72.9°C; IR (KBr, cm^−1^): 3060, 2970, 1738, 1452, 1217, 1015, 750; ^1^H-NMR: 7.96 (d, *J* = 1.32 Hz, 1H), 7.94 (s, 1H), 7.53 (d, *J* = 1.14 Hz, 1H), 7.46–7.32 (m, 4H, ArH), 7.08 (d, *J*= 3.45Hz, 1H), 6.55 (dd, *J* = 3.30, 1.80 Hz, ArH); ^13^C-NMR: 157.79, 156.26, 149.09, 143.48, 134.19, 128.66, 128.20, 126.46, 112.13, 111.83, 108.98; Anal. Calcd. for C_13_H_9_NOS (227.89): C: 68.52, H: 3.98, N: 6.15. Found: C 68.66, H 4.05, N 6.13.

***1i*** [24]: colorless oil; ^1^H-NMR: *δ* 7.96–7.93 (m, 2H), 7.52 (dd, *J* = 3.60, 1.14 Hz, 1H), 7.44–7.32 (m, 5H), 7.05 (dd, *J* = 5.40, 3.60 Hz, 1H); ^13^C-NMR: *δ* 161.15, 155.56, 137.30, 133.95, 128.50, 127.99, 127.60, 127.45, 126.38, 126.26, 111.74. 

***1j***: colorless oil; IR (KBr, cm^−1^): 3034, 1738, 1476, 1365, 1217; ^1^H-NMR: *δ* 7.52–7.48 (m, 2H, ArH), 7.38–7.24 (m, 5H, ArH), 6.78 (s, 1H,), 3.16–3.06 (m, 1H), 1.33 (d, *J* = 6.90 Hz, 6H); ^13^C-NMR: *δ* 166.20, 164.50, 135.85, 133.72, 128.70, 128.57, 126.89, 121.88, 110.26, 30.89, 22.29; Anal. Calcd. for C_14_H_15_NS (229.35): C 73.32, H 6.59, N 6.11. Found: C 73.55, H 6.72, N 6.33.

***1k* [25]**: colorless oil; ^1^H-NMR: *δ* 7.95–7.92 (m, 2H), 7.58–7.55 (m, 2H), 7.47–7.32 (m, 9H); ^13^C-NMR: *δ* 166.76, 156.26, 135.82, 134.52, 134.42, 128.88, 128.75, 128.70, 128.20, 127.12, 126.44, 121.68, 112.09.

***1l* [26]**: white solid, mp: 64.0–65.5 °C (lit. [26] 64°C); ^1^H-NMR: *δ* 7.89–7.85 (m, 2H), 7.44–7.38 (m, 2H), 7.34–7.28 (m, 2H), 2.77 (s, 3H); ^13^C-NMR: *δ* 165.80, 155.22, 134.59, 129.01, 128.69, 127.95, 126.54, 126.34, 112.19, 19.31.

***5a***: colorless oil; IR (KBr, cm^−1^): 2961, 1569, 1509, 1429, 1270, 742; ^1^H-NMR: *δ* 8.45 (t, *J*= 1.75 Hz, 1H), 7.98 (dd, *J* = 7.80, 1.50 Hz, 2H), 7.47 (t, *J*= 7.80 Hz, 1H), 6.90 (d, *J* = 0.72 Hz, 1H), 3.23–3.13 (m, 2H), 1.37 (d, *J* = 6.90 Hz, 12H); ^13^C-NMR: *δ* 166.38, 164.89, 134.65, 129.23, 127.53, 124.37, 111.22, 30.98, 22.32; Anal. Calcd. for C_18_H_20_N_2_S_2 _(328.51): C 65.81, H 6.14, N 8.53. Found: C 65.95, H 6.25, N 8.44.

***5b* [27]**: white solid, mp: 126–126.5 °C; ^1^H-NMR: *δ* 8.14(d, *J* = 7.80 Hz, 2H), 7.86 (t, *J* = 7.80 Hz, 1H), 7.02 (d, *J* = 0.90 Hz, 2H), 2.53 (d, *J* = 0.85 Hz, 6H); ^13^C-NMR: *δ* 165.80, 155.22, 134.59, 128.69, 127.95, 126.34, 112.19, 19.31.

***5c***: white solid, mp: 61.5–62.0 °C; IR (KBr, cm^−1^): 3068, 2926, 1564, 1510, 1498, 1011, 669; ^1^H-NMR: *δ* 8.17 (d, *J*= 7.80 Hz, 2H), 7.85 (t, *J* = 7.80 Hz, 1H), 7.01 (d, *J* = 0.66 Hz, 2H), 3.21–3.12 (m, 2H), 1.37 (d, *J* = 6.90 Hz, 12H); ^13^C-NMR: *δ* 167.68, 165.28, 151.28, 137.80, 119.86, 113.93, 31.10, 22.44; Anal. Calcd. for C_17_H_19_N_3_S_2 _(329.50): C: 61.97, H: 5.81, N: 12.75. Found: C: 61.99, H: 5.85, N: 12.90.

***5d***: colorless oil; IR (KBr, cm^−1^): 2920, 1569, 1485, 1270, 1174, 1072, 731; ^1^H-NMR: *δ* 7.98 (d, *J* = 1.38Hz, 4H), 7.45–7.30 (m, 8H), 3.11 (t, *J* = 7.74Hz, 4H), 2.25–2.20 (m, 2H); ^13^C-NMR: *δ* 174.92, 155.01, 134.67, 128.69, 127.99, 126.42, 113.29, 51.32, 36.64, 16.61; Anal. Calcd. for C_22_H_18_N_2_S_2 _(374.54): C 70.55, H 4.84, N 7.48. Found: C 70.69, H 4.85, N 7.62.
